# Impact of a one-year school-based teacher-implemented nutrition and physical activity intervention: main findings and future recommendations

**DOI:** 10.1186/s12889-020-8351-3

**Published:** 2020-02-19

**Authors:** Carla Habib-Mourad, Lilian A. Ghandour, Carla Maliha, Nancy Awada, Michèle Dagher, Nahla Hwalla

**Affiliations:** 1 Department of Nutrition and Food Sciences, American University of Beirut, Riad El-Solh, Beirut, 1107-2020 Lebanon; 2 Department of Epidemiology and Population Health, American University of Beirut, Riad El-Solh, Beirut, 1107-2020 Lebanon

**Keywords:** Obesity, School nutrition, School-based intervention, Schoolchildren, Lebanon, Overweight, School policy, Sustainability

## Abstract

**Background:**

The aim of the current study is to describe the effectiveness of a school-based intervention when delivered by a non-nutrition specialist (trained schoolteachers) as compared to an expert in nutrition.

**Methods:**

Two trials of the same school-based intervention using the same intervention package were delivered, one by nutritionists and another by trained schoolteachers. The intervention focused mainly on dietary behaviours, as well as physical activity. In both trials, purposively selected schools were randomized to intervention or control groups; students (aged 9–11 years) in both groups were compared at post-test on knowledge and self-efficacy scores, as well as dietary and physical activity behaviours, controlling for their baseline status on the various measures. All analyses accounted for clustering at the school level.

**Results:**

In both trials, a statistically significantly greater improvement was observed for both the knowledge and self-efficacy scores in intervention vs. school students. When the programme was delivered by trained schoolteachers, frequency of breakfast intake was increased, crisps consumption was reduced, but no change in fruit and vegetable consumption was observed (latter increased when delivered by nutrition professionals only). Physical activity did not improve in both trials.

**Conclusion:**

Trained schoolteachers can have a positive impact on students’ dietary behaviours with the appropriate training to ensure they are equipped with the right information, skills, and resources to deliver the programme with the highest fidelity.

**Trial registration:**

ClinicalTrial.gov Identifier: NCT03040271. Retrospectively registered on 2 February 2017.

## Background

Overweight and obesity in children are one of the most difficult global public health challenges of the twenty-first century [1]. Prevention efforts have mainly focused on school-based interventions to provide students with educational information on how to improve diet, increase physical activity, and/or make healthier food choices [2]. School-based interventions have been shown to be effective at significantly improving students’ health-related knowledge and behaviours [3]. Addressing both diet and physical activity has also been shown to be effective in reducing the risk of obesity (BMI) [4] Typically, school-based interventions have been implemented by trained school staff, with regular follow-ups and supervision from the research teams [5–17]. In some cases, however, members of the research team (as pilot studies to assess programme effectiveness) [18], or health professionals such as nutritionists [19], or even a multi-professional team (physician, psychologist, nutritionists and experts in physical activity) have delivered the programmes [20].

The Middle East region is witnessing among the highest rates of obesity globally [21]. While the prevalence of overweight has attenuated over the last years in developed countries, there seem to be continued increase in countries in the Middle East [21, 22]. In Lebanon, a small country in the Eastern Mediterranean region, the prevalence of overweight and obesity have doubled in the past 12 years [22], and in parallel, behavioural risk factors including unhealthy eating habits and low physical activity have also become more prevalent [23]. To this end, a school-based multicomponent intervention focusing on the promotion of healthy eating and active living was pilot-tested in 2009 [24]. The intervention was effective in reducing the purchase and consumption of high energy dense snacks and beverages, and in increasing students’ nutritional knowledge and self-efficacy [24]. The main challenges and lessons learned emanating from this school-based intervention has been also summarized elsewhere [25].

This school-based intervention was then rolled-out by a team of nutritionists in 2010/12 and later by trained schoolteachers in 2012/13. The aim of this paper is to present side-by-side the results of the programme when delivered by non-nutrition specialists (i.e. the trained schoolteachers) and nutritionists. To our knowledge, no study has attempted to present and discuss findings of the same school-based nutrition intervention when delivered by different personnel. School-based interventions that are impactful when delivered by trained school personnel can enhance ownership of the program and ensure its sustainability.

## Method

### Programme implementation

**Fig. 1:**
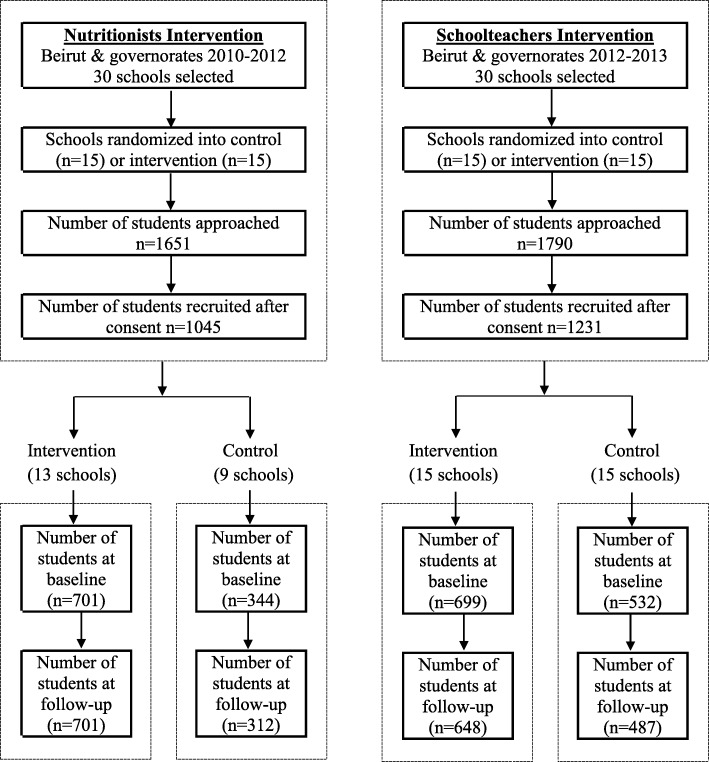
Flow diagram of schools and students’ selection in both interventions

The school-based intervention is developed as a 1 year program. Its implementation by the team of nutritionists took 2 years to cover 30 schools all over Lebanon (Oct 2010- June 2011- Oct 2011- June 2012). Implementation by the trained schoolteachers in 30 schools was carried out during the academic year 2012–2013. In both trials, all students in Grades 4 and 5 (aged 9–11 years) enrolled in the participating schools were invited to participate. The total number of students who agreed to partake in the study is shown in Fig. 1.

### School selection and randomization

The Lebanese Ministry of Education and Higher Education was in charge of the school recruitment process for both trials. Thirty schools were purposively selected for the trial delivered by the nutritionists (each randomized into either intervention or control (15 schools in each group). After collecting consent forms, a total of 22 schools remained in this trial, of which 13 were in the intervention group and 9 were in the control. For the trial delivered by the teachers a new set of 30 schools were purposively selected, then randomized into either intervention or control (15 schools in each group).

### Teacher training

In the second trial delivered by trained teachers, two persons were trained in each participating school in the intervention group: a science teacher and a health educator. The workshops consisted of three full days of training on all programme components and hands-on coaching on all educational activities. A complete toolkit comprised of detailed lesson plans and educational material (posters, pamphlets, booklets…) was shared with the trained teachers.

### Intervention

Students in the intervention schools received the programme components over three consecutive months; in parallel, students enrolled in control schools were receiving their usual curriculum. The intervention specifically targeted obesity-related behaviours in 9–11 year olds including increasing consumption of fruits and vegetables, favouring healthy over energy-dense snacks and drinks, increasing eating breakfast daily, increasing moderate physical activity, and decreasing overall sedentary behaviour.

The intervention was based on the constructs of the Social Cognitive Theory [26], which uses a multilevel approach involving individual behaviour changes and environment modifications to support positive changes at the individual level. The intervention addressed personal-level factors influencing individual behaviour (e.g., knowledge, skills and self-efficacy) as well as environmental-level factors (e.g., modelling and availability). The intervention was comprised of three coordinated modules. First, twelve culturally appropriate classroom sessions using fun and interactive activities were incorporated into the school curriculum and delivered once a week to address the knowledge and self-efficacy determinant, influencing individual behaviour. Second, a family module consisting of meetings, health fairs and information packets sent home including recipes and food samples. Third, a food service intervention targeted the school shops and the lunch boxes sent by the families. Role modelling of significant others and availability of healthy choices at home and school were the main environmental factors addressed by the programme. A detailed description of the intervention components was described previously [27].

### Instrumentation and data collection procedure

All participating students (in intervention and control schools) completed a baseline assessment (pre-test) a week prior to starting the intervention (in the schools randomly assigned to receive the intervention); another post-assessment was conducted, 1 week after completing the intervention (post-test). Both assessments took place in the classrooms.

The questionnaire used in the pre- and post-assessment in both interventions was the one used previously in the original pilot study [24]. It comprised of 3 sections, each assessing a specific primary outcome: (1) dietary behaviours (13 questions); (2) physical activity (10 questions); (3) nutrition knowledge (14 questions) and self-efficacy (9 questions).

The questions on dietary and physical activity behaviours were analysed individually. Questions on dietary behaviours included: purchase and consumption of fruits, crisps, sweetened beverages, and candy bars as snacks as well as questions assessing the frequency of daily consumption of these foods. Categorical variables were recoded as binary to reflect recommended levels of dietary habits, and assess effectiveness of the programme in improving these outcomes. For example, given that the recommendation is to have breakfast daily, the initial question was recoded to reflect daily breakfast versus sometimes/never. For knowledge questions, each response was recoded as either 1 (correct answer) or 0 (for an incorrect answer, or a “don’t know” response), and summed to generate a total score (range: 0–14) reflecting overall knowledge level. The 9 self-efficacy items were also summed into a total score (range 0–18); originally each question was measured on a 3-point Likert scale (0 = not sure, 1 = little sure, 2 = very sure); the higher the score, the better the self-efficacy. The internal consistency (and item-total correlations) of each set of knowledge and self-efficacy items was checked prior to creation of the overall scores; in both cases, the internal consistency was acceptable (Cronbach alpha: 0.66–0.7 at pre assessment and 0.66–0.7 at post assessment) in both interventions.

### Data analysis

Stata MP 13 was used to run mixed effects logistic regression models to compare the intervention and control groups on the selected outcomes of interest at post-test, controlling for baseline levels, accounting for the clustering of students within schools. The critical alpha level was set at 0.05.

## Results

Table 1 presents the results of the dietary knowledge and self-efficacy scores, and as can be seen, at post-test, the scores were statistically significantly higher in the intervention vs. control group students controlling for their baseline measures. This was observed in both trials, although the improvements in dietary knowledge and self-efficacy were greater when the intervention was implemented by the nutritionists than trained schoolteachers.

**Table 1:** Coefficient for change Comparing Knowledge and Self-Efficacy scores in Intervention/Control Groups at Post-Test, Controlling for Baseline Measures

Determinant	Nutritionists trial		Trained Schoolteachers trial	
	β-Coefficient	95% CI	β-Coefficient	95% CI
Knowledge score	2.97	2.68; 3.68	1.45	0.85; 2.04
Self-efficacy score	2.00	1.45; 2.50	0.74	0.28; 1.19

The findings for changes in dietary behaviours and physical activity are presented in Table 2. In the first trial delivered by nutritionists, the odds of daily breakfast intake was the same in both intervention and control groups at post-test when controlling for their baseline breakfast intake (OR: 1.02, 95% CI: 0.56; 1.85); in contrast, when the intervention was delivered by trained schoolteachers, the odds of consuming breakfast daily was twice as high on average among students in the intervention versus control schools at post-test controlling for baseline status (OR: 2.04, 95% CI: 1.34; 3.11).

**Table 2:** Odds Ratios and 95% Confidence Intervals Comparing Intervention/Control Groups on Selected Behaviours at Post-Test

Indicators	Nutritionists trial		Trained Schoolteachers trial	
	Odds ratio	95% CI	Odds ratio	95% CI
Dietary habits				
Daily breakfast intake	1.02	0.56; 1.85	2.04	1.34; 3.11^a^
Intake of fruits (at least twice per day)	1.61	1.19;2.19^a^	1.25	0.85; 1.85
Intake of vegetables (at least once per day)	1.90	1.31;2.75^a^	1.27	0.87; 1.85
Crisps consumption (at least once per day)	0.44	0.23;0.85^a^	0.49	0.30; 0.81^a^
Snacks bought from school shop				
Purchase of soft drinks (yes vs. no)	0.70	0.41; 1.21	0.80	0.31; 2.05
Purchase of crisps (yes vs. no)	0.52	0.29;0.92^a^	0.87	0.34; 2.22
Snacks consumed between meals				
Fruits consumed as snack	1.40	0.94; 2.04	1.42	0.79; 2.53
Crisps consumed as snack	0.85	0.61; 1.19	0.31	0.20; 0.47^a^
After-school physical activities (at least once per week)	1.63	0.83; 3.23	0.51	0.23; 1.13

^a^Significant at *p* < 0.05

Contrary to breakfast intake, significant improvements were observed for recommended levels of fruits (OR: 1.61, 95% CI: 1.19; 2.19) and vegetables (OR: 1.90, 95% CI: 1.31; 2.75) when the intervention was delivered by the nutritionists (Table 2) but not the trained teachers (Table 2). Crisps consumption (at least once per day) was however significantly, and similarly, reduced in both interventions by about 55% (Table 2). No changes were observed for after school physical activity outcomes in both trials.

## Discussion

The present study demonstrates that an effective school-based nutrition intervention could generate promising results if delivered by trained schoolteachers, though the positive impact was not consistent across all dietary outcomes. Although schoolteachers were adequately trained on the programme components and delivery, it is possible that a more intensive training is needed to compensate for the lack of a nutritional background and training. A lack of a solid training or background in nutrition or health promotion techniques has been shown to reduce programme impact and will need to be better addressed in self-sustained school-based intervention programmes [28–30].

While the results were not consistently positive across health outcomes, trained schoolteachers were successful at improving the odds of students’ daily breakfast intake, and reducing their crisps intake. Trained schoolteachers may have been especially motivated to advocate for daily breakfast intake given their own belief that breakfast is linked to improved cognitive performance and classroom behaviour [31], thus the differences seen in breakfast behaviour may be due to regular reinforcement of the messages being provided. In the current study, teachers used experiential learning approaches (cooking and food preparation activities), which have been recently shown in a review article to have the greatest effect on improving children’s eating habits [32]. One enabling factor for reduced crisps consumption in both interventions is perhaps the recent law enforced by the Ministry of Education and Higher Education to stop the sale of high-energy snacks and beverages in school shops. Similar reductions in the consumption of low-nutrient dense foods such as potato crisps, hot dogs and soft drinks have been observed in other studies including schools that had adopted specific nutrition policies regulating the sale of certain food products on campus [33–35]. This stresses the role of public policies in encouraging the availability of healthy food choices in schools to enhance the impact of nutrition education. Our qualitative discussions with the trained school teachers who implemented the program gave us some perspective on potential challenges. For example, unlike breakfast activities, we learnt from our discussions that the fruits and vegetables sessions were not always experiential due to budget constraints that limited bringing fruits and vegetables to class (not the case when the programme was delivered by the nutritionists). This reality further stresses the influential role of situational factors (e.g., available resources) in affecting programme effectiveness beyond programme or staff-relevant factors.

Students’ physical activity did not change or improve in both interventions, which may be explained by external factors such as limited accessibility to extra-curricular activities, be it due to budget constraints, homework overload or the lack of safe and free places for spontaneous physical activity or play, all of which were reported as barriers to improved frequency of after-school sports in previously held focus group discussions as part of the process evaluation of the pilot study [24] Other reviews have shown that the null effect of school-based physical activity interventions on children’s moderate to vigorous physical activity may be due to interventions not reaching target populations as intended. Authors concluded that further assessments of intervention fidelity are required [36]. Increasing the number of physical education sessions per week, at school, may be a more appropriate goal for schools in low to middle-income countries. Other researchers have noted that change in physical activity may necessitate more targeted individual behavioural interventions which was not the case in our study [37].We acknowledge that our study has limitations. Dietary behaviors and physical activity were self-reported and thus were subjectively assessed, and are prone to reporting error. Another limitation, is the reduced number of physical education sessions and the absence of sports experts in both trials, which may have affected the improvement in students’ physical activity levels as nutritionists are probably not best suited to deliver active living components. While this study is the first to describe the findings of two trials delivering the same intervention package, it did not directly assess the difference in the impact of the intervention when delivered by the nutritionists or the schoolteachers. Finally, the baseline dietary and physical activity behaviours, as well as knowledge and self-efficacy scores were comparable between students who were lost to follow-up and those with complete data, within and across intervention and control groups; thus, it is unlikely that any differential misclassification bias was introduced.

## Conclusion

Overall, the present study demonstrates the potential for trained schoolteachers to deliver effective school-based nutrition interventions provided they are equipped with a solid training in nutritional information.

Making a positive impact would necessitate structural changes that go beyond teacher training and include school administration’s financial plan in supporting the implementation of such in-class programmes, as well as commitment to improving structural determinants of health including the physical environment (presence of play areas to increase physical activity) and school policies (availability of healthy food choices). In that realm, trained schoolteachers can become advocates and agents of change for a more sustainable, long-term health and nutrition promotion programme within schools.

Finally, it is recommended that an expert nutritionist works with the school staff to monitor and ensure adequate implementation of the programme during its early stages, and maintains a consultant role to the school.

## Data Availability

The datasets generated and/or analysed during the current study are available from the corresponding author on reasonable request.

## References

[1] Musaiger AO (2011). Overweight and obesity in eastern mediterranean region: prevalence and possible causes. J Obes.

[2] Waters E, de Silva-Sanigorski A, Hall BJ, Brown T, Campbell KJ, Gao Y, Armstrong R, Prosser L, Summerbell CD (2011). Interventions for preventing obesity in children. Cochrane Database Syst Rev.

[3] Scherr RE, Linnell JD, Dharmar M, Beccarelli LM, Bergman JJ, Briggs M, Brian KM, Feenstra G, Hillhouse JC, Keen CL (2017). A Multicomponent, School-Based Intervention, the Shaping Healthy Choices Program, Improves Nutrition-Related Outcomes. J Nutr Educ Behav.

[4] Brown T, Moore THM, Hooper L, Gao Y, Zayegh A, Ijaz S, Elwenspoek M, Foxen SC, Magee L, O'Malley C (2019). Interventions for preventing obesity in children. Cochrane Database Syst Rev.

[5] Caballero B, Clay T, Davis SM, Ethelbah B, Rock BH, Lohman T, Norman J, Story M, Stone EJ, Stephenson L (2003). Pathways: a school-based, randomized controlled trial for the prevention of obesity in American Indian schoolchildren. Am J Clin Nutr.

[6] Manios Y, Kafatos A, Mamalakis G (1998). The effects of a health education intervention initiated at first grade over a 3 year period: physical activity and fitness indices. Health Educ Res.

[7] Manios Y, Moschandreas J, Hatzis C, Kafatos A (1999). Evaluation of a health and nutrition education program in primary school children of Crete over a three-year period. Prev Med.

[8] Manios Y, Moschandreas J, Hatzis C, Kafatos A (2002). Health and nutrition education in primary schools of Crete: changes in chronic disease risk factors following a 6-year intervention programme. Br J Nutr.

[9] Saksvig BI, Gittelsohn J, Harris SB, Hanley AJ, Valente TW, Zinman B (2005). A pilot school-based healthy eating and physical activity intervention improves diet, food knowledge, and self-efficacy for native Canadian children. J Nutr.

[10] Gortmaker SL, Peterson K, Wiecha J, Sobol AM, Dixit S, Fox MK, Laird N (1999). Reducing obesity via a school-based interdisciplinary intervention among youth: planet health. Arch Pediatr Adolesc Med.

[11] Reed KE, Warburton DE, Macdonald HM, Naylor PJ, McKay HA (2008). Action schools! BC: a school-based physical activity intervention designed to decrease cardiovascular disease risk factors in children. Prev Med.

[12] Gentile DA, Welk G, Eisenmann JC, Reimer RA, Walsh DA, Russell DW, Callahan R, Walsh M, Strickland S, Fritz K (2009). Evaluation of a multiple ecological level child obesity prevention program: Switch®what you Do, View, and Chew. BMC Med.

[13] Spiegel SA, Foulk D (2006). Reducing overweight through a multidisciplinary school-based intervention. Obesity (Silver Spring).

[14] Fernandes PS, Bernardo Cde O, Campos RM, Vasconcelos FA (2009). Evaluating the effect of nutritional education on the prevalence of overweight/obesity and on foods eaten at primary schools. J Pediatr.

[15] Amaro S, Viggiano A, Di Costanzo A, Madeo I, Baccari ME, Marchitelli E, Raia M, Viggiano E, Deepak S, Monda M (2006). Kaledo, a new educational board-game, gives nutritional rudiments and encourages healthy eating in children: a pilot cluster randomized trial. Eur J Pediatr.

[16] Taylor RW, McAuley KA, Barbezat W, Strong A, Williams SM, Mann JI (2007). APPLE project: 2-y findings of a community-based obesity prevention program in primary school age children. Am J Clin Nutr.

[17] Danielzik S, Pust S, Landsberg B, Muller MJ (2005). First lessons from the Kiel obesity prevention study (KOPS). Int J Obes.

[18] Kelder S, Hoelscher DM, Barroso CS, Walker JL, Cribb P, Hu S (2005). The CATCH kids Club: a pilot after-school study for improving elementary students’ nutrition and physical activity. Public Health Nutr.

[19] Kain J, Uauy R (2004). Albala, Vio F, Cerda R, Leyton B: school-based obesity prevention in Chilean primary school children: methodology and evaluation of a controlled study. Int J Obes Relat Metab Disord.

[20] Damon S, Dietrich S, Widhalm K (2005). PRESTO--prevention study of obesity: a project to prevent obesity during childhood and adolescence. Acta Paediatr Suppl.

[21] Ng M, Fleming T, Robinson M, Thomson B, Graetz N, Margono C, Mullany EC, Biryukov S, Abbafati C, Abera SF (2014). Global, regional, and national prevalence of overweight and obesity in children and adults during 1980–2013: a systematic analysis for the global burden of disease study 2013. Lancet.

[22] Nasreddine L, Naja F, Chamieh MC, Adra N, Sibai AM, Hwalla N (2012). Trends in overweight and obesity in Lebanon: evidence from two national cross-sectional surveys (1997 and 2009). BMC Public Health.

[23] Naja F, Hwalla N, Itani L, Baalbaki S, Sibai A, Nasreddine L (2015). A novel Mediterranean diet index from Lebanon: comparison with Europe. Eur J Nutr.

[24] Habib-Mourad C, Ghandour LA, Moore HJ, Nabhani-Zeidan M, Adetayo K, Hwalla N, Summerbell C (2014). Promoting healthy eating and physical activity among school children: findings from health-E-PALS, the first pilot intervention from Lebanon. BMC Public Health.

[25] Habib-Mourad C, Ghandour LA (2015). Time to Act: Lessons Learnt from the First Pilot School-Based Intervention Study from Lebanon to Prevent and Reduce Childhood Obesity. Front Public Health.

[26] Bandura A (1986). Social foundations of thought and action: a social cognitive theory.

[27] Habib-Mourad C, Moore H, Nabhani-Zeidan M, Hwalla N, Summerbell C (2014). Health-E-PALS: promoting healthy eating and physical activity in Lebanese school children – intervention development.

[28] Wang D, Stewart D (2013). The implementation and effectiveness of school-based nutrition promotion programmes using a health-promoting schools approach: a systematic review. Public Health Nutr.

[29] Nutbeam D (1992). The health promoting school: closing the gap between theory and practice. Health Promot Int.

[30] Nutbeam D (1995). Exposing the myth. What schools can and cannot do to prevent tobacco use by young people. Promot Educ.

[31] Radcliffe B, Ogden C, Welsh J, Carroll S, Coyne T, Craig P (2005). Queensland school breakfast project: a health promoting schools approach. Nutr Diet.

[32] Dudley DA, Cotton WG, Peralta LR (2015). Teaching approaches and strategies that promote healthy eating in primary school children: a systematic review and meta-analysis. Int J Behav Nutr Phys Act.

[33] Mullally ML, Taylor JP, Kuhle S, Bryanton J, Hernandez KJ, MacLellan DL, McKenna ML, Gray RJ, Veugelers PJ (2010). A province-wide school nutrition policy and food consumption in elementary school children in Prince Edward Island. Can J Public Health.

[34] Budd GM, Volpe SL (2006). School-based obesity prevention: research, challenges, and recommendations. J Sch Health.

[35] Cullen KW, Watson K, Zakeri I (2008). Improvements in middle school student dietary intake after implementation of the Texas public school nutrition policy. Am J Public Health.

[36] Love R, Adams J, van Sluijs EMF (2019). Are school-based physical activity interventions effective and equitable? A meta-analysis of cluster randomized controlled trials with accelerometer-assessed activity. Obes Rev.

[37] Tymms PB, Curtis SE, Routen AC, Thomson KH, Bolden DS, Bock S, Dunn CE, Cooper AR, Elliott JG, Moore HJ (2016). Clustered randomised controlled trial of two education interventions designed to increase physical activity and well-being of secondary school students: the MOVE project. BMJ Open.

